# Framework for Supporting Adolescent Peer Leaders: A Pilot Using Text Messaging in a School-Based Substance Use Prevention Program

**DOI:** 10.1007/s10935-019-00545-4

**Published:** 2019-03-02

**Authors:** Anthony R. Pisani, Peter A. Wyman, Mariya Petrova, Emily Judd, Karen Schmeelk-Cone, Phyo Thiha, Kunali Gurditta

**Affiliations:** 1 Department of Psychiatry, School of Medicine and Dentistry, University of Rochester, Rochester, NY 14642 USA; 2 Department of Pediatrics, School of Medicine and Dentistry, University of Rochester, Rochester, NY 14642 USA; 3 Department of Public Health Sciences, Miller School of Medicine, University of Miami, Coral Gables, FL 33124 USA; 4 Department of Computer Science, University of Rochester, Rochester, NY 14627 USA

**Keywords:** Implementation support, Peer leaders, Text messaging, Pilot, Substance use prevention

## Abstract

Training peer leaders (PLs) as implementation agents is a state-of-the-art approach in prevention, but the field lacks frameworks for providing support. Text messaging, a powerful tool for direct intervention, may be useful in this regard. We introduce a conceptual framework for engaging, retaining, and educating adolescent PLs and conduct a pilot test of this framework using text messages for delivery to middle school PLs in a new, peer-led substance use prevention program. Fifty eighth-graders were recruited as PLs. We used a newly-developed framework to create text messages to strengthen peer leaders’: (a) mission, agency, and team identity; (b) connection to adult mentors; (c) content knowledge and application to their own lives; and (d) preparation for prevention activities. Thirty-four texts were sent to PLs over 4 months. PL replies and participation were recorded to track engagement. Forty-one PLs (71%) received texts and completed baseline and post-program surveys. Parents and school staff completed post-program questionnaires. Eighty-five percent of PLs responded to at least one text message. Response rates for specific messages varied from 22 to 56%. Students were most likely to reply to texts about preparation for their own prevention activities in the school. Ninety-five percent of PLs said they read messages even when they did not reply. Eighty-three percent of PLs said the messages helped them accomplish their mission. PLs reported that they wanted to receive messages in the future. PL attendance had very little variability in two of the three schools, but replies to texts were associated with better attendance in one school. Our study provides a framework for supporting adolescent peer leaders in a network intervention. Automated text messaging supporting middle school PLs was feasible, engaging, and well-received. Texting activity was associated with participation in school-based activities. Future priorities include systematically varying text support to determine its true effect on implementation and on involvement by less engaged PLs.

## Background

Grounded in social learning (Bandura, 1986) and diffusion of innovation (Rogers, 2003) theories, peer leader programs train socially influential members of a group to adopt desired healthy behaviors and encourage others in their social networks to do the same. Peer leader programs reduce tobacco and HIV risk behaviors (Campbell et al., 2008; Latkin, 1998; Sikkema et al., 2000), and show promise for promoting healthy coping norms and help-seeking behavior in adolescent suicide prevention (Wyman et al., 2010). Recently, we reported that training and retaining more peer leaders (Wyman et al., 2010) predicted greater diffusion of a suicide prevention program (Wyman et al., 2015). However, conceptual frameworks for supporting adolescent peer leaders are lacking.

To address this need, we developed a framework drawing on well-tested constructs from self-determination theory (Ryan & Deci, 2000) and findings from our own qualitative interviews with student leaders in an existing school-based suicide prevention program (Wyman et al., 2010). The framework focuses on enhancing peer leaders’: (a) mission, team identity, and sense of agency; (b) connection to adult mentors in school; (c) content knowledge and application in peer leaders’ own lives; and (d) preparation for prevention activities. We piloted text messages based on the framework with middle school peer leaders delivering substance use prevention programming.

This framework guided the development of automated, interactive text messages designed to supplement school-based meetings with adult mentors. Text messaging is effective for promoting health behavior change (Cole-Lewis & Kershaw, 2010; Head et al., 2013; Mason et al., 2016; Suffoletto, 2012) but its use as a tool for supporting peer opinion leaders is largely unexplored. One project used text messages for implementation support to malaria case managers (Jones et al., 2012; Zurovac et al., 2011) but these messages were atheoretical: that is, they consisted of basic reminders and inspirational proverbs.

We pilot-tested text messages with peer leader groups from three middle schools to determine (a) engagement and acceptance; (b) association between engagement (i.e., text replies) and intervention participation; and (c) parent and staff perceptions.

## Method

### Participants

Fifty-eight peer-nominated eighth grade students (mean age 13.8 years) across three rural/exurban (semirural area beyond the suburbs of a city) New York middle schools participated as peer leaders in a new, substance use prevention program, representing 12.1% of eighth grade population (school range 8–17%). The schools followed a two-step process for identifying peer leaders. First, all eighth grade students in the school completed a questionnaire asking them to identify 2–3 students “who are seen as ‘leaders,’ i.e., students whose voice is heard by other students at school.” School administrative staff reviewed the nominated students and added additional students with the aim of increasing the diversity and reach of peer leaders into different social groups across the school. Of the 58 peer leaders, 15 either did not have cell phones or chose not to participate in the texting portion of the program. Two peer leaders signed up to receive text messages but left the school prior to completing the study. The final group of 41 received texting support and participated in the current study. Excluding gender, where peer leaders were 64% female compared to a school population close to 50%, demographics of peer leaders were very close to those of the schools as a whole (race: 92% White, 2% African American, 1% Asian, 1% Native American; Hispanic ethnicity: 5%). In addition to the students, 18 parents (72% female) of peer leaders completed an anonymous post-program questionnaire, as did 76 school staff members (72 teachers, 1 administrator, 3 pupil support services staff; 71% female). The University of Rochester IRB approved the protocol.

### Pilot Intervention and Text Messages for Peer Leaders

In collaboration with the Partnership for Drug Free Kids, we tested a peer-led school-based intervention for eighth grade students, conceptually linked to the national *Above the Influence* (ATI) campaign (Carpenter & Pechmann, 2011; Scheier et al., 2011; Slater et al., 2011). During an initial 3-h training, peer leaders were coached to: (1) identify positive personal and relational resources that help them stay “true to themselves”; (2) identify negative social influences; (3) adopt personal resistance strategies; and (4) learn accurate information about peer drug and alcohol use (social norming). Trained adult mentors facilitated eight to eleven 30-min meetings over 3 months to help peer leaders plan and conduct school-wide campaigns aimed at introducing their peers to the ATI concepts and modeling their use of the concepts.

Using the framework described above, we composed 34 texts to be sent to peer leaders over 4 months (2–3 times/week); 11 requested a keyword or free text response and five contained a link to view a related webpage or video. Text messages were sent using an automated, interactive texting system developed at the University of Rochester. As a safety precaution, staff reviewed student text responses within 24 h of sending the messages. In the unlikely event that students texted concerning content (e.g., material touching on suicide, abuse, or bullying), staff had a protocol for alerting investigators and school partners.

### Measures

#### Parent Acceptability

As part of enrollment and consent procedures, we asked parents to provide their child’s cell phone number, and also gave students the opportunity to decide for themselves if they wished to receive texts. In terms of our pilot evaluation, we considered parent and student willingness as an indicator of acceptability.

#### Peer Leader Surveys and Participation

Peer leaders completed post-program participation surveys. Participation in meetings and messaging activities was monitored and reported by ATI coaches.

#### Parent/Staff Feedback

Post-program questionnaires administered to parents and school staff assessed their perception of the value of the messages and their interest in receiving messages themselves in a future program.

#### Student Interaction with Text Messages

We recorded student replies to messages and the number of times students followed links to websites or videos.

#### Data Availability

Supplementary files containing deidentified data from all sources (parent, staff, peer leader), categorized by content, are available from the corresponding author.

## Results

### Interaction With the Implementation Text Messages

Peer leaders consistently engaged with text messages. Thirty-five of 41 students (85.4%) replied to at least one message, and 30 (73.2%) responded to three or more. The proportion responding to any interactive message ranged from 22 to 56.1%, with students responding to a mean of 3.5 (*SD *= 2.54) messages. Examples of messages sent and PL responses are shown in Table 1. Comparing responses to the different domains of the peer leader support framework, PLs replied most frequently to messages about Preparation for Prevention Activities. As shown in Table 2, 83% of students replied to at least one message in this domain, compared with 68% for Connection to ATI Coach and School, 66% for Concept Knowledge and Personal Application, and 44% for Enhancing Mission and Team Identity. The raw mean number of responses we received for messages from this domain was 3.3, compared with 1.4, 1.9 and 0.6 for the other domains, respectively, although the difference was not statistically significant.

**Table 1:** Framework for supporting peer leaders using text messaging

Implementation goal	Message themes	Types of text messages to communicate themes	Examples of text messages sent to peer leaders
Enhancing mission, team identity, and sense of agency	Peer leaders’ participation is valued and needed in making ATI relevant to their school and their group of friends Peer leaders are part of a bigger movement Peer leaders have expertise and opinions that matter	Informational texts about how peers influence each other Feedback from school-based activities Examples of work from teams at other schools National videos (abovetheinfluence.com) Requests for feedback about the program, and what could make it better, more relevant Questions about what messages, themes and strategies will influence their particular friends	Don’t forget—students and teachers at your school nominated you to be a Peer Leader because others look up to you. You can define what’s cool! Welcome and congrats for joining Above the Influence (ATI) at (specific school)! You are part of a national teen movement! :) abovetheinfluence.com Congratulations on completing your first challenge! How do you think it went?
Connection to school advisors and ATI mentors	Peer leaders’ ATI advisors and mentors care about them, support their group being effective, and are dedicated to the cause	Encouraging messages on behalf of advisors and mentor	Your adult advisors said you did an amazing job last Friday! You put up 901 stickers!
Content knowledge and personal application	Peer leaders grow as people, friends, and leaders ATI is personal to peer leaders and their friends Rising above negative influences is endorsed by most students	Requesting replies for personal examples of: Staying true to you Rising above negative pressures Positive supports Social norming polls/Q&A	If a kid comes up to you in the hall and asks, “What is ATI?” You would say… How does an adult help you stay true to you? A) I trust and can talk to them B) They’re real w/me and encourage me C) I can be myself w/them D) Not sure Did you know? 96% of 8th graders at (specific school) don’t think drinking alcohol will make them more popular
Preparation for school-based prevention activities	Getting ready for meetings and activities makes them more exciting and effective ATI is fun	Goal reminders, preparatory videos, meeting reminders Links to photos of PLs having fun/being silly	Students at other ATI schools say they were nervous to present in front of their friends, but it paid off! How do you feel about the upcoming presentations? Text us something funny or memorable from your ATI meeting this week. We’ll pick a couple and share them back to you (no names included!)….

**Table 2:** Text responses by category

	Total *n* messages	*n* asked for response	Raw mean asked response	Mean by asked response^+^	% responded to at least one (asked for response)
Enhancing mission and team identity	8	2	0.58 (.741)	0.29 (.370)	43.9
Connection to ATI coach and school	7	4	1.41 (1.264)	0.35 (.316)	68.3
Concept knowledge and personal application	20	5	1.88 (1.749)	0.38 (.350)	65.9
Preparation for prevention activities	20	9	3.29 (2.358)	0.37 (.262)	82.9

^+^Mean # responses/total possible texts within those where we asked for a response

Boys and girls responded equally well. When text messages included internet hyperlinks, between 10 (24.4%) and 41 (100%) students clicked links. There was no evidence of drop-off or fatigue. Student replies were appropriate, generally on-point, and no safety concerns were detected. Table 3 shows illustrative texts and responses. A few student responses reflected minor misunderstandings about ATI or the PL role (e.g., PLs help students with problems or bullying). Knowledge of this hidden misconception gave adult mentors and research staff the opportunity to revisit program concepts.

**Table 3:** Results of peer leader, parent, and school staff surveys

Participants	Questions	% Agree/Strongly agree
Peer Leader I would be interested in receiving…	I read the messages even when I didn’t reply	95.1
	The messages kept me focused on our mission	82.9
	…links to videos	81.0
	…invitations to share advice and experiences anonymously	92.9
	…questions that get you thinking about how you handle pressures	95.1
	…quotes and stories from students facing the same stressors you face	90.5
	…short games like “choose your own adventure” related to ATI	87.8
Parents	Occasional text messages to peer leaders is a good way to support their mission and involvement	88.9
	I would be interested in receiving occasional text messages designed for parents of students involved in ATI	83.3
School Staff	Substance abuse prevention should incorporate internet use and text messaging	94.5
	I would be interested in receiving occasional text messages designed for those involved in ATI	59.5

*ATI* is an abbreviation for Above the Influence

### Perceptions of Implementation Support

Text messaging was well-received by peer leaders, parents, and staff, as displayed in Table 3. Peer leaders found the interactive text messaging helpful and were interested in receiving an even wider variety of messages in the future. Thirty-two students (78%) reported that the message frequency was “about right,” two students (5%) felt there were too many, and seven students (17%) felt there were too few. Most PLs (> 90%) were interested in receiving texts with quotes or stories from students under similar stressors, questions that made them think about how to handle stressors, and invitations to share advice and experiences anonymously. PL attendance had minimal variability in two of three schools, but in the school with variation, replying to text messages was correlated with attendance (*r* = .77, *p* = .005).

Parents and school staff strongly supported Internet use and text messages. A high proportion (83%, *n* = 41) of parents provided their student’s cell phone number. Of the 18 parents (44% of invited) who responded to the survey, 89% viewed texting as a good way to support the PL mission and involvement and 83% wished to receive texts themselves. Staff viewed texting as an important part of substance abuse programming and about half expressed interest in receiving messages.

## Discussion

This study provides both a preliminary framework for supporting adolescent peer leaders as prevention implementation agents and evidence that it is feasible to use this framework to support students via automated, interactive text messages. Positive, strength-based messages were designed to support peer leaders’ identification and personal application of intervention concepts, connection with adult mentors, and participation in activities. Most parents and middle school students agreed to receive messages from this prevention program. Peer leaders engaged actively with text messages, especially those that pertained to supporting their school-based activity—an encouraging finding when juxtaposed with the association between interaction with messages and peer leader attendance in the one school where there was variability in attendance. Peer leaders reported that receiving 1–2 messages per week was the right frequency. Peer leaders viewed the messages as helpful to their mission, which was a core goal of the framework we developed. Parents and staff members were enthusiastic about texting to support peer leaders. Most parents (of the 35% of parents who responded) and about half of staff members were interested in receiving prevention-oriented text messages themselves.

Our next step is to address the limitations and opportunities this small pilot presents. Going forward, it will be important to quantify the impact on peer leaders’ concept uptake, retention, participation, and effectiveness by systematically varying text support and content, and determining which domains of the framework are most critical to emphasize. Because peer leader retention is important for program effectiveness (Wyman et al., 2015), the notable association we found between text activity and peer leader attendance particularly merits further research. One key goal is to test whether texting increases engagement among peer leaders who are less engaged and more peripheral in the school social network.

Practical considerations remain. For example, research staff in this small pilot, who were in close touch with adult mentors in each school, set a customized schedule of automated messages to synchronize with school-based meetings and activities. Scaling up this level of customization to multiple schools would be difficult. However, our texting application is easy to use and many teachers already use group texting programs (e.g., programs such as Remind; http://www.webcitation.org/71THISFnD) so this is a notable but surmountable challenge. We conclude that text messaging using a concept-driven framework holds promise for supporting primary prevention program implementation by adolescent peer leaders.
